# General Law on Personal Data Protection and applicability to Nursing

**DOI:** 10.1590/0034-7167-2023-0126

**Published:** 2023-12-04

**Authors:** Fernanda Cirne Lima Weston, Ana Carolina Ballesteiros Paglioli, Mônica Weston Mesquita

**Affiliations:** I Pontifícia Universidade Católica do Rio Grande do Sul. Porto Alegre, Rio Grande do Sul, Brazil; II Universidade Federal do Rio Grande do Sul. Porto Alegre, Rio Grande do Sul, Brazil; III Santa Casa de Misericórdia de São Paulo. Porto Alegre, Rio Grande do Sul, Brazil.

**Keywords:** Jurisprudence, Nursing, Computer Security, Medical Informatics, Confidentiality, Jurisprudencia, Enfermería, Seguridad Computacional, Informática Médica, Confidencialidad, Jurisprudência, Enfermagem, Proteção de Dados, Informática Médica, Confidencialidade

## Abstract

**Objectives:**

to reflect on the impacts of the General Personal Data Protection Law on Nursing practice.

**Methods:**

reflection article, through the intentional collection of materials relating to the topic.

**Results:**

legislation regulates confidentiality, processing and data sharing, requiring institutional protection measures. The nursing team is responsible for acting preventively, both in care and in the management role, in order to avoid the misuse of the patient’s personal data. The law allows academic research to be carried out as long as the purpose is clear, data collection occurs with an explicit purpose and data is anonymized.

**Final Considerations:**

although the General Personal Data Protection Law requires greater care in relation to data processing, it is established on precepts of good faith and respect for the rights of the individual, concepts aligned with the nursing code of ethics.

## RESUMO

**Objetivos:**

refletir sobre os impactos da Lei Geral de Proteção de Dados Pessoais na prática da enfermagem.

**Métodos:**

artigo de reflexão, por meio da coleta intencional de materiais referentes ao tema.

**Resultados:**

a legislação regulamenta o sigilo, o tratamento e o compartilhamento dos dados, exigindo medidas de proteção institucionais. À equipe de enfermagem cabe agir preventivamente, tanto na assistência quanto no papel gerencial, a fim de evitar o mau uso dos dados pessoais do paciente. A lei permite a realização de pesquisas acadêmicas desde que a finalidade esteja clara, que a coleta de dados ocorra com um propósito explícito e que seja realizada a anonimização dos dados.

**Considerações Finais:**

apesar da Lei Geral de Proteção de Dados Pessoais exigir maiores cuidados em relação ao tratamento dos dados, ela é estabelecida em preceitos de boa-fé e em respeito aos direitos do indivíduo, conceitos alinhados ao código de ética da enfermagem.

## RESUMEN

**Objetivos:**

reflexionar sobre los impactos de la Ley General de Protección de Datos Personales en la práctica de enfermería.

**Métodos:**

se trata de un artículo reflexivo llevado a cabo mediante una recolección intencional de materiales referentes al tema.

**Resultados:**

la legislación regula la confidencialidad, el tratamiento y la puesta en común de los datos, exigiendo medidas institucionales de protección. Corresponde al equipo de enfermería actuar de forma preventiva, tanto en la atención como en la gestión, para evitar el uso indebido de los datos personales de los pacientes. La ley permite la investigación académica siempre que el propósito sea claro, los datos se recojan con un fin explícito y se anonimicen.

**Consideraciones Finales:**

aunque la Ley General de Protección de Datos de Carácter Personal exige un cuidado mayor con relación al tratamiento de los datos, se basa en preceptos de buena fe y respeto de los derechos del individuo, conceptos que están en consonancia con el código deontológico de la enfermería.

## INTRODUCTION

In times of virtuality, personal data is a projection, a continuity of the human being. Personal data refers to information that relates to an identified or identifiable person; with the use of the internet - and especially social networks - this data takes on different formats, such as photos, images and opinions)^(1)^. Through this exposure, the identification of the individual is facilitated, making them vulnerable to coercion and manipulation.

Internationally, there is recognition of the importance of protecting this information. Faced with the occurrence of scandals involving data leaks for the purposes of political manipulation and private interests, several countries, including those belonging to the European Union, have developed standards and legislation that regulate the processing of data^(2)^. In the health sector, this movement is no different, since, although computerized records and care systems are positive for the management and quality of care provided, it is recognized that they make data more vulnerable to leaks and inappropriate uses^(3)^.

Given the need to protect personal information, the General Personal Data Protection Law (LGPD) was created^(1)^. This law was published in 2018 and aims to standardize the processing of personal data - especially those available online - in order to protect the individual’s right to intimacy and privacy and implement sanctions for non-compliance. The health sector is also covered by this legislation, as the LGPD allows data processing to occur for health procedures provided by professionals, services and/or authorities^(1)^.

Nursing, specifically, deals daily with sensitive data, recorded as recommended by the Federal Nursing Council (COFEN), valuing the maintenance of health, the best provision of care and the protection of the professional themselves^(4)^. Possessing this information entails the need for nurses to remain up to date and aware of the standards that deal with the protection of users and services. Such awareness on the part of the professional is fundamental, as it reduces the risks to the individuals cared for as well as to the nurse’s own career.

Considering the relevance of the topic, this article intends to bring brief lines about the ways in which nursing adequately complies with the LGPD in the performance of its activities, examined here from three aspects: assistance, scientific research and management activities.

Google Scholar databases using the terms “General Law for the Protection of Personal Data” and “LGPD” in May 2023, they were not identified by the authors articles on the topic applied specifically to nursing practice. Therefore, one must reflect on its possible impacts and applications in clinical practice, as well as on the conduct to be adopted by organizations and the nursing team itself, in order to protect the individual.

## GENERAL LAW ON PROTECTION OF PERSONAL DATA AND NURSING

For a better understanding and reflection on the LGPD, it is important to explain the basic concepts on which it is based. The LGPD is a law that aims to regulate the “processing” of data, defining this action as

any operation carried out with personal data, such as those relating to collection, production, reception, classification, use, access, reproduction, transmission, distribution, processing, archiving, storage, elimination, evaluation or control of information, modification, communication, transfer, diffusion or extraction^(1)^;

Within this concept, special attention is given to data considered sensitive, which can be processed and disclosed only in situations described by law. When talking about sensitive data, it refers to information such as religion, ethnicity, sexual orientation, political and/or philosophical positioning, participation in unions, biometrics, health and genetic information - often present in nursing records^(1)^.

**Figure 1:**
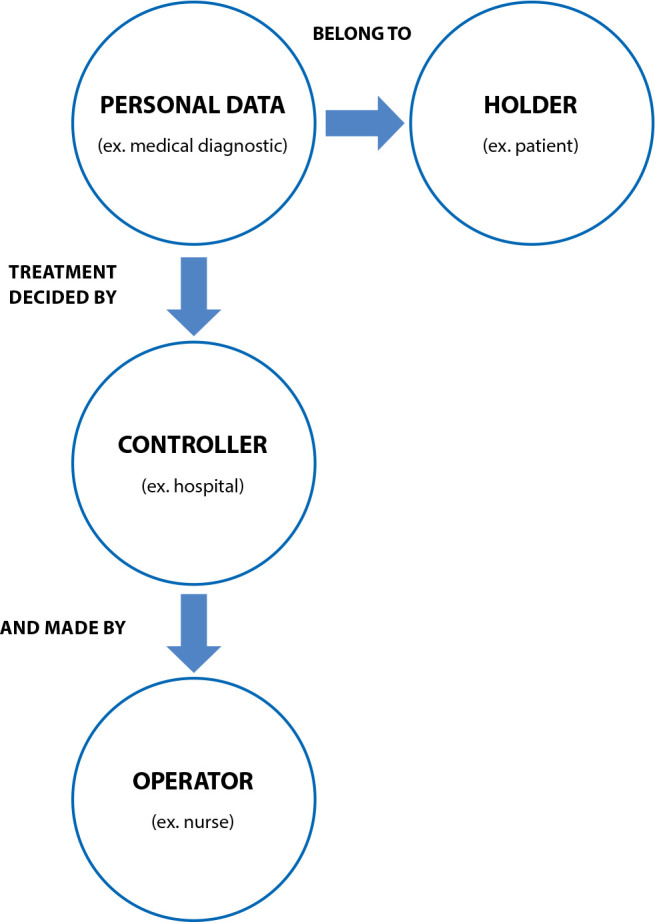
Relationships between parts of the General Personal Data Protection Law

According to the law, the processing of personal data takes place based on the relationship between three parties: the “holder”, who is the owner of the data, which is the patient; the “controller”, that is, the person/institution responsible for making decisions regarding the processing of data; and the “operator”, the person who processes data on behalf of the “controller” (Figure 1)^(1)^.

Nursing’s responsibility for processing sensitive data changes depending on the environment in which these professionals perform their duties. Generally, the nursing professional assumes the role of “operator” in relation to personal data; however, if a nurse is the technical manager or performs his or her duties in a private practice, he or she will be assuming the role of “controller”.

Regardless of the role that the professional performs in relation to “personal data”, be it “operator” or “controller”, the handling of sensitive data of the “holder”, or that is, the patient. Thus, compliance with the LGPD and the nursing team’s own regulations, including the Code of Professional Ethics and the regulations of the Federal Nursing Council (COFEN), is a routine practice and is essential for the good performance of the profession^(4-5)^.

## APPLICABILITY IN NURSING CARE

The nursing team works in different health contexts, whether in outpatient settings, such as primary care, or in the context of hospital admissions. In all environments where nursing operates, data is generated, mainly through health records in medical records^(1)^.

In the last decade, through computerization in health, electronic medical records have been more widely used and have even been recommended by international councils and organizations^(4)^. The use of electronic medical records presents relevant advantages and the most complete alignment with the LGPD. The legislation authorizes the processing of personal data to protect the patient’s life, as well as allowing the same information to be accessed from multiple locations. However, it must be considered that this makes data more vulnerable to inappropriate access and use.

In patients’ electronic medical records, access restrictions are common only to professionals hired by the institution, a conduct consistent with what is recommended by the LGPD. However, the law goes further, encouraging that only health professionals involved in patient care can record and access their health data. The addition of this restriction would not only protect the patient but would also protect the healthcare institution and the nursing professional themselves. If an incident occurs, the legal assessment of severity will consider all measures proven to be used to prevent access by unauthorized third parties^(1)^. Therefore, not only the establishment of individual users and passwords in electronic medical records should be encouraged, but also access restrictions related to the professional’s position in the institution.

Despite requiring greater care, the LGPD does not provide for any changes to nursing records. The Federal Nursing Council (COFEN) requires that records contain the individual’s sensitive personal data, covering information such as the patient’s family and social context, nursing diagnoses and behaviors adopted by the team^(4)^. Recognizing the importance of these data for personalized and effective care in different health areas, these behaviors must be maintained, and the information that must appear in the nursing record must not be changed.

It is considered that the LGPD is consistent with the requirements of COFEN. It is based on good faith and the principles of purpose, adequacy, necessity, free access to data subjects, data quality, transparency, security, prevention, non-discrimination and accountability on the part of the data holder^(1)^. For nursing, these principles do not differ from those already recommended by the profession itself, with the LGPD, therefore, aligned with the Code of Ethics for Nursing Professionals^(5)^.

However, the law brings improvements in relation to the care of personal data, establishing that everyone involved in the processing of data must protect this information through protection measures, both technically and administratively. It is up to the nursing team to act preventively, in order to avoid unauthorized access to the patient’s personal data. The team must also avoid situations of misuse of data, such as leaks and uses for purposes other than health.

It is important to reflect on the possible consequences for the nursing team if there is non-compliance with the LGPD, consequences that include civil liability before the health institution or before the patient, via legal proceedings; administrative order before the National Data Protection Authority (NDPA), according to the criteria of Resolution no. 04 of February 34, 2023 and also an ethical disciplinary order for failure to comply with the duty of secrecy^(6)^. In other words, the consequences for the professional could range from compensation for damage caused to fines, warnings and even sanctions from the Council, depending on the type of link the team has in relation to the “controller” of personal data, and the path of liability - civil or administrative - of the nursing professional.

The law reinforces the importance of confidentiality, a general rule in health relationships, and imposes punishments for treatment not authorized by law. Therefore, it is the professional’s duty to protect the patient’s privacy, an obligation clearly recognized by the nursing team, whether through the LGPD or through compliance with the Code of Ethics.

## REFLECTIONS IN SCIENTIFIC RESEARCH

Nursing is a complex science whose decisions and technical conduct must be based on evidence^(5)^. In this sense, carrying out new research is extremely important for the qualification of care, and the use of the internet and other electronic media is an important tool in data collection and analysis.

The LGPD recognizes that data may be processed for academic purposes; allows the processing of data when there is consent by the holder and “when necessary to meet the legitimate interests of the controller or a third party, except in the case where fundamental rights and freedoms of the holder prevail that require the protection of personal data”^(1)^. Legitimate interests include academic research based on the principles of law, reason and justice and which do not violate the freedoms of the individual being researched.

When carrying out research with human beings, the application of the Informed Consent Form (ICF) is now mandatory, in which the holder’s consent is necessary to respect ethical precepts^(7)^. Resolution No. 510 of April 7, 2016 also guaranteed the right to secrecy and privacy of the research participant, who has the right to protect their personal data^(7)^. However, retroactive research is common in academic nursing, in which data from medical records is collected without the individual’s consent.

The LGPD allows these surveys to be carried out as long as their purpose is clear, that data collection occurs with an explicit purpose and that data is anonymized, in accordance with Resolution No. 466 of December 12, 2012, which already predicted that research should have a real possibility of answering the proposed research question and that participant confidentiality should always be maintained^(8)^. In the case of developing new technologies and products present in academic nursing research, this information is also applicable. The requirements of the LGPD also meet what is ethically recommended in nursing research - as the objective of the research must be explicit, relevant to nursing practice and consistent with the chosen methods - and also adds the need to protect the data generated.

NDPA has already expressed its support for the processing of personal data for academic purposes and for carrying out studies by research bodies. The NDPA also recognizes that the LGPD itself allows dialogue between the right to privacy and the guarantees of freedom of expression and pluralism of ideas in the academic environment. Therefore, this weighing of values made by the legislator himself justifies the legal permissiveness contained in the LGPD, which excludes the application of the law in the case of data processing for academic purposes, as long as the protection of intimacy and privacy is maintained^(9)^.

## MANAGEMENT PRACTICE AND DATA LEAKS

The nurse’s role is not limited to the direct care of patients and, within the roles assigned to this professional, management practice is included. This performance requires characteristics such as entrepreneurship, organization and planning capacity, allowing the professional to participate in the decision-making of their organization^(10)^. When performing this role, nurses are required to know different aspects of the LGPD, in order to assist the healthcare institution in planning and executing measures to prevent leaks and protect data.

According to the law, the processing of personal data will be irregular when it fails to comply with legislation or when it does not provide the security that the holder can expect. The issue of leaks and breach of confidentiality seems to be framed under the “security” hypothesis, in which, following the norm, the “controller” and “operator” are responsible - *here* the nursing team is often included.

The “security” that the holder can expect in the processing of data, according to the LGPD, will be assessed according to the way in which the processing is carried out, the result, the risks that are reasonably expected and, finally, the processing techniques that were employed at the time in question. Therefore, the choice of suitable software for health records that have mechanisms to prevent access by third parties must be encouraged by the nursing professional manager, even if this falls under the responsibility of the controller.

Furthermore, it is up to nursing to ensure the protection of patient and health institution data, through actions such as not sharing personal passwords and not opening external *links* on work equipment, always establishing measures to prevent possible harm. use of this information. An example of misuse is data leakage - the incidence of which has been increasing in recent years - in which patients’ personal information can be held hostage, demanding a sum of money from the institution in exchange for maintaining its confidentiality^(3)^.

In addition to participating in choosing and encouraging safer software with more restrictive access, the nurse manager can also develop educational activities aimed at guiding the institution’s professionals about the LGPD. Topics such as confidentiality, safe conduct on the internet and ways to prevent data leaks must be addressed for greater security of the institution and the patient.

## FINAL CONSIDERATIONS

The recent publication of the LGPD impacted the functioning of health institutions, leading to the emergence of doubts regarding its effects on management, education and research. For nursing, as it frequently deals with sensitive and non-anonymized personal data, this impact could not be different.

The LGPD came as a reaffirmation of the protection of privacy, a fundamental patient right. Just like professional secrecy, the law is nothing more than a tool established to protect an individual’s privacy. Both follow the same logic: the use of personal data is intended to benefit its holder, that is, the patient, and its sharing must be conditioned on prior authorization and exceptionally authorized in cases provided for by legislation.

The LGPD requires greater care in relation to data processing and is established on principles of good faith and respect for the rights of the individual. These concepts are already common and closely related to the nursing code of ethics. It is up to these professionals to understand current legislation, valuing the protection of data and the users to whom it belongs.

Therefore, it is essential that future research evaluate tools and methods for teaching and learning professionals about the LGPD, as it is a multidisciplinary content, not commonly covered in nursing degrees. It is also suggested that research be carried out that includes in detail the application of this legislation for each work environment of these professionals, as this article aimed to reflect on a general way of complying with the LGPD. Other research may also be carried out, such as reporting on the development of training for nursing professionals on this topic, as well as articles evaluating possible data vulnerabilities in the work environment of these professionals. Through new research, it is expected that professionals will recognize the applicability of this legislation, in order to protect both the institution and the patients who use it.
